# Implementation of a Hospital-Based Screening and Treatment Program for Unhealthy Alcohol Use

**DOI:** 10.1007/s11606-026-10473-9

**Published:** 2026-05-14

**Authors:** Jananie Ramesh, Christopher Moriates, John Embers, Alanna Boulton, Nicole Kluz, Rachel Holliman, Frances Ibarra, Ohenewaa Dede-Bamfo, Patrick Chang, Mary Velasquez, Michael Pignone

**Affiliations:** 1 Department of Internal Medicine, Dell Medical School at The University of Texas at Austin, Austin, TX USA; 2 Veterans Affairs Greater Los Angeles Healthcare System, Los Angeles, CA USA; 3 Department of Medicine, University of California, Los Angeles, Los Angeles, CA USA; 4 CommUnity Care Health Centers, Austin, TX USA; 5 Central Health, Travis County Health District, Austin, TX USA; 6 Department of Population Health, Dell Medical School at The University of Texas at Austin, Austin, TX USA; 7 The University of Texas at Austin, Steve Hicks School of Social Work, Austin, TX USA; 8 Department of Medicine, Duke University, Durham, NC USA

**Keywords:** alcohol use, brief intervention, hospital medicine, quality improvement

## Abstract

**Background:**

Screening and treatment for unhealthy alcohol use (UAU) and alcohol use disorder (AUD) are recommended but underutilized, particularly in hospital settings.

**Objective:**

To determine whether a comprehensive screening and treatment protocol for UAU in hospitalized patients can help reduce alcohol use.

**Design:**

Quality improvement study at single-center safety-net hospital.

**Participants:**

27,914 patients were admitted from 9/2022 to 9/2024, and 18,146 (65.0%) were screened with AUDIT-PC, with 1085 (6.0%) screening positive (≥ 5). Substance use navigators (SUNs) identified 257 additional patients with UAU through referral methods.

**Intervention:**

SUNs approached eligible patients and conducted full AUDIT and brief behavioral counseling. Patients with AUDIT ≥ 12 were offered medications for AUD (MAUD) during hospitalization and at discharge.

**Main Measures:**

Intervention delivery, 30-day return hospital encounters (readmission or emergency department), and change in AUDIT score among high-risk patients.

**Key Results:**

Of the 1342 intervention-eligible patients, 81% were men, and the mean age was 51 years. 54% were Caucasian, 30% Hispanic/Latinx, and 9% Black. 17% were unhoused, and 18% were uninsured. Of these 1342, 800 (59.6%) patients engaged in the intervention. The mean AUDIT score was 15 (median 14, IQR 8–21). For the 489 (61.1%) patients with AUDIT ≥ 12, 231 (47.2%) were started on medication before discharge. For the 126 patients with repeat AUDIT (25.8%), the mean reduction was 14.2 points (95% CI: −15.4, −12.2). For those with AUDIT ≥ 12, 128 (26.2%) patients had a return hospital encounter within 30 days of discharge. Patients without return encounters had significantly larger reductions in AUDIT score than those who had return encounters (mean reduction −16.2 vs −9.1 points (difference −7.1, 95% CI: −3.1, −11.0)).

**Conclusions:**

UAU screening and risk-stratified treatment (including pharmacotherapy) can be delivered in diverse hospitalized patients, and were accompanied by short-term changes in alcohol risk score among patients reached for follow-up.

**Supplementary Information:**

The online version contains supplementary material available at https://doi.org/10.1007/s11606-026-10473-9.

## BACKGROUND

Unhealthy alcohol use (UAU) is a common, under-recognized, and undertreated condition with significant consequences for cardiovascular health, liver health, and cancer risk.^1–7^ Approximately 20% of adults in the USA report binge drinking in the past month, and almost 10% are considered to have alcohol use disorder (AUD).^8^ AUD is even more common among hospitalized patients.^9^ Moreover, the consequences of UAU disproportionately affect marginalized and socioeconomically disadvantaged populations.^10–12^

Hospitalization offers a unique opportunity to identify UAU and initiate treatment since patients may be motivated to make changes in their health behaviors. Hospitalized patients with UAU tend to have more severe disease and other co-morbidities, and are often admitted due to consequences of UAU.^13–16^ Identification and treatment of UAU is an opportunity to prevent significant morbidity, especially in vulnerable populations who may face barriers in accessing healthcare.

Screening, brief intervention, and referral to treatment (SBIRT) protocols have proven effective in primary care and emergency department settings for increasing short-term abstinence and reducing alcohol-related harm in patients with UAU.^17–23^ However, the efficacy of brief interventions alone has not been consistently demonstrated on hospitalized patients with AUD,^24–29^ and limited studies have tracked the important metric of successful outpatient care linkage after discharge.^30,31^ As such, the screening, treatment initiation, and referral (STIR) model has been designed to add initiation of evidence-based medications at the index visit, with evidence of improved efficacy in addressing tobacco use, opioid use, and alcohol use disorders in emergency departments, and offers promise for inpatient intervention.^32–34^

A few studies looking at medication initiation at discharge have demonstrated trends towards decreased readmissions and ED visits.^35–40^ However, medications for AUD (MAUD) are rarely utilized inpatient or outpatient, despite their effectiveness and inclusion in guideline-based therapy for the treatment of UAU.^41–46^ Furthermore, to our knowledge, no study has effectively combined a structured brief intervention with medication initiation for hospitalized patients with UAU.

Based on an assessment identifying gaps in our local care system, our aim was to implement a comprehensive clinical protocol for UAU in hospitalized patients, examine uptake at all stages, and assess for short-term reductions in alcohol use after discharge. We identified patients with UAU, performed risk stratification to identify likely AUD, provided an evidence-based brief intervention on decreasing alcohol use, tailored based on severity of disease, incorporated MAUD initiation to eligible patients, and provided outpatient linkage and follow-up.

## METHODS

We developed a clinical protocol for a safety-net teaching hospital in Austin, Texas, to identify and treat admitted patients with UAU, and collected data from 9/2022 to 9/2024. This report was prepared in accordance with the SQUIRE 2.0 guidelines for quality improvement reporting.

The 240-bed hospital is a Level 1 trauma center with a diverse population that admits over 15,000 patients a year, with four acute care floors and surgical, medical, and neurocritical ICUs. Nurses completed the AUDIT-PC, a 5-question screening tool, in the electronic medical record (EMR) for admitted patients. The screening tool allows default zero scoring without mandatory question completion, which can result in misclassification of incomplete screens as negative. Prior to this project, this alcohol screening did not systematically trigger targeted intervention, and few patients received comprehensive AUD care. There were no pre-existing addiction medicine inpatient services available, so we aimed to empower existing prescribers and recruit key non-physician providers to facilitate intervention delivery.

The hospital partners with a large FQHC (federally qualified health center) clinic that provides outpatient addiction medicine services. This clinic had previously developed an ambulatory-based grant-funded program for UAU identification and treatment,^47^ creating an opportunity to introduce an analogous clinical service in the hospital setting.

Funding was provided through the Cancer Prevention and Research Institute of Texas and Texas Health and Human Services. This quality improvement project aimed to provide evidence-based services and was deemed to be a non-human subjects research project by the Institutional Review Board.

For this project, we collaborated with hospital information technology to receive a daily list of all newly admitted patients, their admission diagnoses, and AUDIT-PC scores. Two licensed master social workers were recruited as substance use navigators (SUNs) to deliver the intervention described below. Further details regarding SUN training, screening tools, and workflows are provided in Appendix A.

### Intervention

On weekdays, SUNs identified intervention-appropriate patients with UAU by reviewing daily admission lists for positive (≥ 5) AUDIT-PC scores or alcohol-related admission diagnoses. Patients could also be referred to SUNs via direct consult. For patients who screened positive, SUNs administered the full AUDIT to confirm and stratify the severity of UAU and then delivered a severity-tailored intervention.^48^

- Patients with AUDIT scores < 12 are considered low risk for moderate-severe AUD. For these patients, the SUN completed a brief behavioral intervention (BI) and placed a referral to primary care (SBIRT model). Given constrained resources and evidence supporting BI effectiveness in lower-risk UAU, additional outreach and outcome measurement were not prioritized for this group.^49^
- Patients with AUDIT scores ≥ 12 were considered at high risk of moderate-severe AUD. For these patients, SUNs delivered BI and additionally provided counseling regarding MAUD. When patients expressed interest and had no contraindications, SUNs recommended MAUD initiation to the inpatient care team prior to discharge (STIR model). SUNs coordinated primary care follow-up and also facilitated referrals to outpatient addiction care and community-based recovery resources as appropriate.

Our program aimed to focus resources on patients that were most likely to benefit from consideration of MAUD initiation. We used an AUDIT score cutoff of 12 to facilitate identification of patients who could benefit from more intensive therapy. SUNs documented encounters in the EMR and recorded intervention data in a HIPAA-compliant Research Electronic Data Capture (REDCap) database. Additional details regarding BI content, referral processes, and documentation workflows are provided in Appendix A.

### Outcomes Assessment

At 4–6 weeks after discharge, SUNs attempted to contact high-risk patients (initial AUDIT ≥ 12) to complete an outcomes assessment. Follow-up included a repeat AUDIT using a modified time frame (“since discharge”), assessment of outpatient appointment attendance, MAUD adherence, and interval emergency department visits or readmissions. Detailed follow-up procedures are described in Appendix A.

For all patients with an AUDIT ≥ 12, an inpatient chart review was completed to assess 30-day emergency department visits or readmissions within the hospital system.

For all patients with an AUDIT ≥ 12 and linkage to our partner FQHC clinic system, an outpatient chart review was conducted to assess follow-up appointment attendance and medication adherence.

### Analysis

We analyzed the population of patients with positive AUDIT-PC screens and stratified patients by brief intervention completion, as well as AUDIT score. Demographic characteristics were compared between groups using odds ratios. For patients with AUDIT scores ≥ 12 who completed the follow-up assessment, we compared baseline and follow-up AUDIT scores, mean difference, and score change distribution. We also conducted analyses to evaluate associations between patient characteristics, intervention uptake, outcomes response, and 30-day return encounters. Further details regarding statistical methods are provided in Appendix A.

## RESULTS

**Figure 1:**
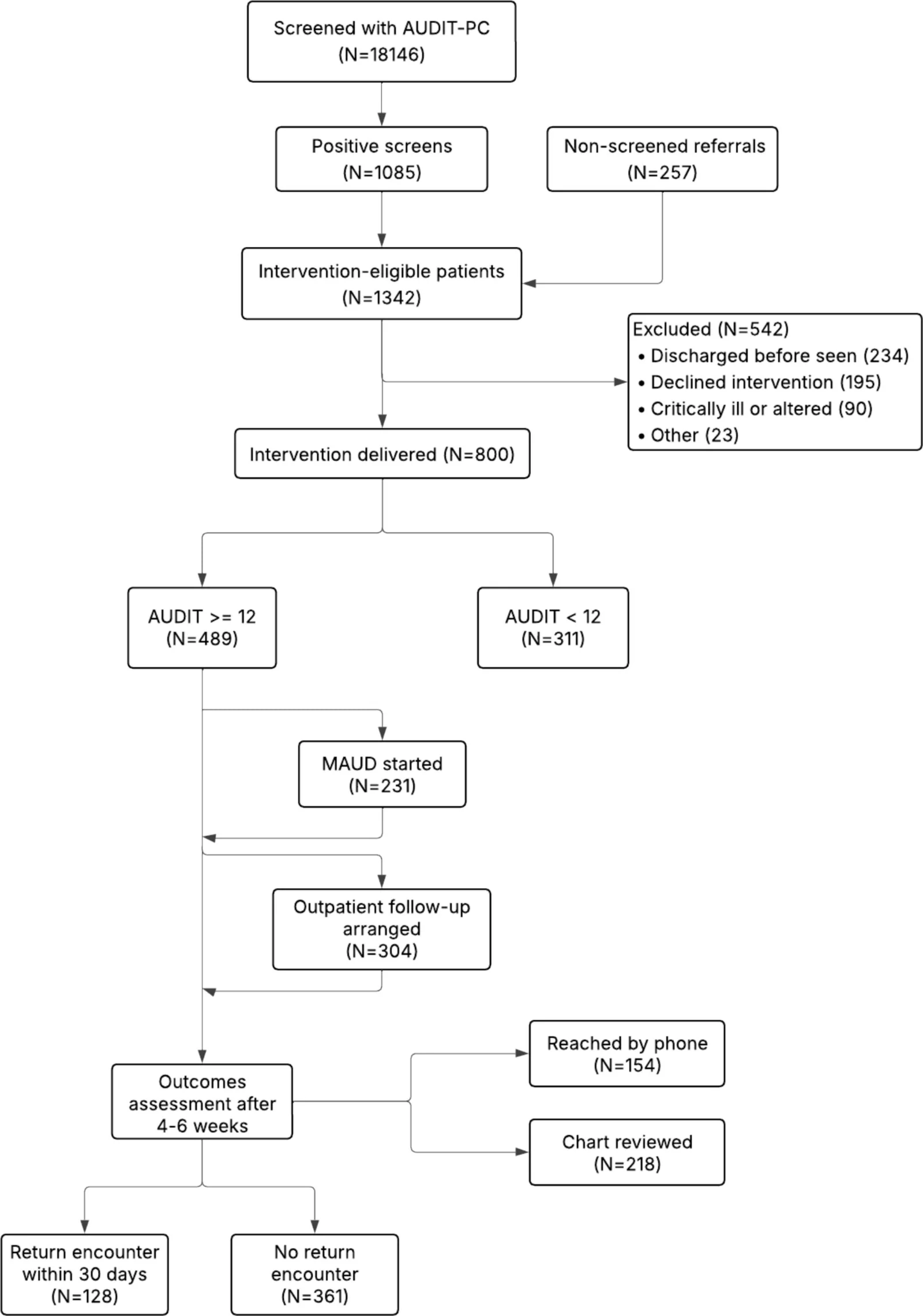
Patient flow diagram.

From 9/2022 to 9/2024, 27,914 patients were admitted to the hospital. Nursing screened 18,146 patients (65.0%) with the AUDIT-PC, and 1085 (6.0%) screened positive (≥ 5). Contributing factors to incomplete screens included inability to administer screens to critically ill patients, limitations on screen completion in the emergency room-boarded patients, and lack of familiarity with substance use workflows among providers. SUNs identified 257 additional patients with UAU through other methods (direct consults or an alcohol-related reason for admission). Thus, 1342 patients were identified with high likelihood of UAU and were eligible for intervention (Fig. 1).

Characteristics of the intervention-eligible patients are shown in Table 1. Eight hundred of these 1342 patients (59.6%) received the brief intervention. Patients who did not receive the brief intervention (*n* = 542) were discharged before being seen (*n* = 234, 43.1%), declined participation (*n* = 195, 36.0%), were critically ill (*n* = 90, 16.6%), or were unable to participate for other reasons (*n* = 23, 4.2%) (Fig. 1). Most of those not seen were admitted and discharged over the weekend or on holidays, when we did not have SUN coverage. Of the 1018 patients who were approached, 78.6% (800) engaged in the intervention.

**Table 1:** Demographics of Intervention-Eligible Patients

	**Patients who completed behavioral intervention**	**Patients who did not complete behavioral intervention**	**Total**	**Odds ratios**
	(*N* = 800)	(*N* = 542)	(*N* = 1342)	OR (95% CI)
**Sex**				
Male	649 (81.1%)	433 (79.9%)	1082 (80.6%)	Reference
Female	149 (18.6%)	109 (20.1%)	258 (19.2%)	0.91 (0.69, 1.20)
N/A	2 (0.3%)	0	2 (0.1%)	
**Age**				
< 50 years	390 (48.8%)	247 (45.6%)	637 (47.5%)	Reference
≥ 50 Years	410 (51.3%)	295 (54.4%)	705 (52.5%)	0.89 (0.72, 1.11)
Mean ± SD	50.5 ± 13.7	52.1 ± 15.8	51.2 ± 14.6	
**Self-identified race**				
White or Caucasian	415 (51.9%)	313 (57.7%)	728 (54.2%)	Reference
Hispanic or Latinx	286 (35.8%)	116 (21.4%)	402 (30.0%)	**1.88 (1.45, 2.45)**
Black or African American	75 (9.4%)	48 (8.9%)	123 (9.2%)	1.18 (0.80, 1.75)
Other or unknown*	24 (3.0%)	65 (12.0%)	89 (6.6%)	0.28 (0.17, 0.45)
**Housing status**				
Housing/temporary housing	661 (82.6%)	446 (82.3%)	1107 (82.5%)	Reference
No housing	139 (17.4%)	96 (17.7%)	235 (17.5%)	0.97 (0.73, 1.30)
**Insurance status**				
Commercial	165 (20.6%)	113 (20.8%)	278 (20.7%)	Reference
Uninsured	155 (19.4%)	83 (15.3%)	238 (17.7%)	1.28 (0.89, 1.83)
MAP (Medical Access Program)	204 (25.5%)	84 (15.5%)	288 (21.5%)	**1.66 (1.17, 2.36)**
Medicaid	51 (6.4%)	18 (3.3%)	69 (5.1%)	**1.94 (1.10, 3.57)**
Medicare	75 (9.4%)	46 (8.5%)	121 (9.0%)	1.12 (0.72, 1.74)
Other or unknown	150 (18.8%)	198 (36.5%)	348 (25.9%)	0.52 (0.38, 0.72)
**Admission reason**				
Admitted due to alcohol use	473 (59.1%)	202 (37.3%)	675 (50.3%)	**2.42 (1.94, 3.03)**

^*^Other category includes the following: American-Indian, Asian, Native Hawaiian, and other

The patients who engaged in additional assessment and intervention (*n* = 800) were more likely to be Hispanic/Latinx (OR: 1.88, 95% CI: 1.45–2.45), have MAP (county-based medical access program coverage) or Medicaid insurance (OR: 1.66, 95% CI 1.17–2.36; OR: 1.94, 95% CI 1.10–3.57), and were more likely admitted due to alcohol use (OR: 2.42, 95% CI 1.94–3.03) (Table 1).

For the 800 patients who received assessment and intervention, mean AUDIT score was 15 (median 14, IQR 8–21), and 489 patients (61.1%) scored ≥ 12, signifying higher likelihood of AUD. These 489 patients were more likely to have MAP insurance (OR: 1.8, 95%CI: 1.13–2.90) and be admitted for reasons related to alcohol use (OR: 6.64, 95% CI: 4.86–9.14), but less likely to be < 50 years (OR: 0.68, 95% CI 0.51–0.90). Over 75% (*n* = 372) of these patients were admitted due to alcohol use.

### MAUD initiation

Patients with an AUDIT score ≥ 12 received education about MAUD. Out of these 489 patients, 231 (47.2%) were started on medication before discharge, mostly oral naltrexone (*n* = 217, 90.8%). Eighty patients (16.3%) were ineligible for naltrexone due to severe hepatic dysfunction or concurrent use of opioids, and 130 patients (26.6%) declined medication. Another 33 patients (6.7%) were not started on medication due to provider-related reasons.

### Follow-up referrals

Out of the 489 patients with AUDIT ≥ 12, the SUNs coordinated primary care referrals for 304 (62.2%) patients. Two hundred eighty-one (92.4%) of these patients were referred to our FQHC partner clinics, of which some were already established patients.

Of the 185 patients who did not receive a SUN-coordinated primary care appointment, 89 patients (48.1%) had an established primary provider outside of our FQHC partners and preferred to arrange their own appointment. Fifty-nine patients (31.7%) were going to a skilled nursing or rehabilitation facility, 27 patients (14.6%) declined the appointment, and 10 patients (5.4%) did not receive an appointment for other reasons (Appendix B, Table 1).

### Outcomes assessment

We completed follow-up phone calls for 154 of 489 (31.5%) patients with AUDIT ≥ 12, and 126 of these patients (81.8%) completed a repeat AUDIT assessment. Patients who were reached by phone were more likely to be stably housed and commercially insured than those who were not reached. No major differences were noted in initial AUDIT score between responders and non-responders, although non-responders were somewhat more likely to have been admitted due to alcohol use.

Among those reached, mean reduction in AUDIT was 14.2 points (95% CI: −15.4, −12.2) (Table 2). One hundred ten patients (87.3%) decreased their AUDIT score by one point or more, 13 patients (10.3%) had an unchanged score, and 3 patients (2.4%) had a higher score. The distribution of AUDIT score changes can be seen in Appendix B, Table 3; with 72% of patients achieving a score reduction of > 9 points. Of note, 42 patients (33.3%) had stopped drinking entirely. There were no major differences in mean score change across demographic categories, though patients admitted for an alcohol-related reason had a larger mean score reduction (−15.0 vs −11.9 points) (Appendix B, Table 2).

Outpatient chart reviews were completed for an additional 218 patients (44.6%) who could not be contacted by phone. One hundred seventeen patients (23.9%) were not reached by phone and could not be chart reviewed as they did not follow at our partner FQHCs (Table 2). Of the 372 (76.1%) patients with a follow-up phone call and/or chart review data, 193 (51.9%) attended their follow-up appointment. Of these 372 patients with follow-up data, 191 patients (51.3%) had a MAUD prescription at discharge, and 181 patients (48.7%) were not prescribed MAUD. Of those with MAUD prescriptions, 86 patients (45.0%) reported continued adherence to medication at follow-up and 32 patients (16.8%) reported non-adherence, and for 73 patients (38.2%), adherence to MAUD was unknown or not documented (Table 2).

**Table 2:** Outcomes Assessment

Follow-up data for patients with AUDIT ≥ 12	Phone interview (*N* = 154)	Outpatient chart review (*N* = 218)	Total patients with follow-up data (*N* = 372)	Patients with no follow-up data (*N* = 117)
Average initial AUDIT score	20.6	20.2	20.3	19.9
Median initial AUDIT (IQR)	20 (15.3–24.8)	20 (15.0–24.0)	20 (15.0–24.0)	19 (14.0–24.0)
Admitted due to alcohol use	119 (77.3%)	159 (72.9%)	278 (74.7%)	94 (80.3%)
Patients with follow-up AUDIT	126 (81.8%)	0		
Average follow-up AUDIT score	6.4	N/A		
Median follow-up AUDIT (IQR)	4.0 (0.0–8.0)	N/A		
Average AUDIT score difference	−14.2	N/A		
**Appointment attendance**				
Attended follow-up care	92 (59.7%)	101 (46.3%)	193 (51.9%)	
Attended new appointment	66	95	161	
Followed with own provider	26	6	32	
Did not attend follow-up care	25 (16.2%)	91 (41.7%)	116 (31.2%)	
Unknown	25 (16.2%)	9 (4.1%)	34 (9.1%)	
Not appointed	12 (7.8%)	17 (7.8%)	29 (7.8%)	
**Medications**				
# of patients prescribed medication	80 (51.9%)	111 (50.9%)	191 (51.3%)	
Adherent to medication	47 (58.8%)	39 (35.1%)	86 (45.0%)	
Non-adherent	22 (27.5%)	10 (9.0%)	32 (16.8%)	
Unknown	11 (13.8%)	62 (55.9%)	73 (38.2%)	
# of patients not started on medication	74 (48.1%)	107 (49.1%)	181 (48.7%)	

### Readmissions

For the 489 patients with AUDIT ≥ 12, 128 patients (26.2%) had a return hospital encounter (ED visit or readmission) (Table 3).

**Table 3:** Demographics of 30-Day Return Encounters

	**Patients with return encounters**	**Patients with no return encounters**	**Total**	**Unadjusted odds ratio**	**Adjusted odds ratio**
	(*N* = 128)	(*N* = 361)	(*N* = 489)	OR (95% CI)	OR (95% CI)
**Sex**					
Male	103 (80.5%)	294 (81.4%)	397 (81.2%)	Reference	Reference
Female	23 (18.0%)	67 (18.6%)	90 (18.4%)	0.98 (0.57, 1.63)	1.25 (0.70, 2.20)
N/A	2 (1.6%)	0	2 (0.4%)		
**Age**					
< 50 years	53 (41.4%)	201 (55.7%)	254 (51.9%)	Reference	Reference
≥ 50 years	75 (58.6%)	160 (44.3%)	235 (48.1%)	**1.78 (1.18, 2.68)**	1.52 (0.95, 2.42**)**
Mean ± SD	50.9 ± 12	48.2 ± 12.5	48.9 ± 12.4		
**Self-identified race**					
White or Caucasian	71 (55.5%)	184 (51.0%)	255 (52.1%)	Reference	Reference
Hispanic or Latinx	40 (31.3%)	140 (38.8%)	180 (36.8%)	0.74 (0.47, 1.15)	0.76 (0.45, 1.26)
Black or African American	15 (11.7%)	24 (6.6%)	39 (8.0%)	1.62 (0.79, 3.24)	1.15 (0.52, 2.48)
Other or unknown*	2 (1.6%)	13 (3.6%)	15 (3.1%)	0.4 (0.06, 1.49)	0.60 (0.09, 2.38)
**Housing status**					
Housing/temporary housing	79 (61.7%)	320 (88.6%)	399 (81.6%)	Reference	Reference
No housing	49 (38.3%)	41 (11.4%)	90 (18.4%)	**4.84 (3.00, 7.88)**	**3.74 (2.17, 6.53)**
**Insurance status**					
Commercial	16 (12.5%)	98 (27.1%)	114 (23.3%)	Reference	Reference
Uninsured	20 (15.6%)	92 (25.5%)	112 (22.9%)	1.33 (0.65, 2.76)	1.44 (0.68, 3.12)
Medical Access Program	59 (46.1%)	104 (28.8%)	163 (33.3%)	**3.47 (1.91, 6.62)**	**2.05 (1.02, 4.26)**
Medicaid	15 (11.7%)	19 (5.3%)	34 (7.0%)	**4.84 (2.05, 11.55)**	**3.56 (1.44, 8.89)**
Medicare	12 (9.4%)	35 (9.7%)	47 (9.6%)	2.10 (0.89, 4.87)	1.43 (0.57, 3.51)
Other or unknown	6 (4.7%)	13 (3.6%)	19 (3.9%)	2.83 (0.89, 8.33)	2.52 (0.75, 7.86)
**Admission reason**					
Admitted due to alcohol use	91 (71.1%)	281 (77.8%)	372 (76.1%)	0.70 (0.45, 1.11)	0.79 (0.48, 1.32)

^*^Other category includes the following: American-Indian, Asian, Native Hawaiian, and other

Patients with a return encounter were more likely to be > 50 years of age (OR: 1.78, 95% CI: 1.18–2.68), unhoused (OR: 4.84, 95% CI: 3–7.88), and have Medicaid or MAP funding (OR: 4.48, 95% CI: 2.05–11.55; OR: 3.47, 95% CI: 1.91–6.62) (Table 3). On multivariate analysis, unhoused status and insurance type were independently associated with return encounters (Table 3).

There were no significant differences in initial AUDIT score, MAUD initiation (OR: 0.98, 95% CI: 0.51–1.72), or whether the patient received an outpatient referral (OR 1.28, 95% CI: 0.84–1.97) between patients with and without a return encounter. There were also no significant differences in whether the patient attended their follow-up appointment, or whether they were adherent with MAUD (Table 4).

**Table 4:** Follow-up Data for Patients With and Without Return Encounters

	Patients with ED visits (*N* = 57)	Patients with readmissions (*N* = 71)	Total patients with return encounters (*N* = 128)	Patients without return encounters (*N* = 361)
Initial admission due to alcohol	40 (70.2%)	51 (70.2%)	91 (71.1%)	281 (77.8%)
Re-encounter related to alcohol	34 (59.6%)	42 (59.2%)	76 (59.4%)	N/A
Patients with multiple re-encounters	14 (24.6%)	39 (54.9%)	53 (41.4%)	N/A
Number of distinct encounters	75	128	203	N/A
Average # of encounters per patient	1.32	1.80	1.59	N/A
Average # of days before re-encounter	11.8 days	11.5 days	11.6 days	N/A
**AUDIT scores**				
Initial AUDIT Score	20.7	19.4	20.0	20.3
# of patients with follow-up AUDIT	8 (14.0%)	24 (33.8%)	32 (25.0%)	94 (26.0%)
Average follow-up AUDIT score	11.6	10.3	10.6	5.0
Average score difference	−9.0	−9.1	−9.1	−16.2
**Outpatient referrals**				
Appointment made before discharge	38 (66.7%)	47 (66.2%)	85 (66.4%)	219 (60.7%)
Attended appointment	20 (52.6%)	23 (48.9%)	43 (50.6%)	118 (53.4%)
Did not attend appointment	10 (26.3%)	21 (44.7%)	31 (36.5%)	79 (35.7%)
Unknown	8 (21.1%)	3 (6.4%)	11 (12.9%)	22 (10.0%)
No appointment made before discharge	19 (33.3%)	24 (33.8%)	43 (33.6%)	142 (39.3%)
**Medications for AUD**				
Total patients with MAUD started	31 (54.4%)	36 (50.7%)	67 (52.3%)	179 (49.6%)
Adherence to medication	11 (35.5%)	15 (41.7%)	26 (38.8%)	60 (33.5%)
Not taking medication	2 (6.5%)	5 (13.9%)	7 (10.4%)	25 (14.0%)
Unknown	18 (58.1%)	16 (44.4%)	34 (50.7%)	94 (52.5%)
MAUD not started	26 (45.6%)	35 (49.3%)	61 (47.7%)	182 (50.4%)

Patients without a return encounter (ED or hospital), however, had significantly larger reductions in AUDIT score than those who had return encounters, with a mean reduction of −16.2 vs −9.1 points (difference −7.1, 95% CI: −3.1, −11.0).

## DISCUSSION

This project implemented a screening and risk-stratified treatment protocol for hospitalized patients to identify and treat UAU, including use of MAUD, and facilitated connection to outpatient care. The intervention was delivered with high fidelity to a diverse, underserved population at a safety-net hospital. Although several studies have described the efficacy of brief intervention and MAUD initiation separately, to our knowledge, no previous initiatives have evaluated a combined, risk-stratified intervention of brief counseling, MAUD initiation, and tailored, coordinated outpatient referrals for high-risk hospitalized patients. We also employed outcomes assessment through follow-up phone calls and chart reviews to assess the fidelity of the intervention. We observed robust short-term reductions in alcohol use among those assessed, with the degree of alcohol use reduction inversely correlated with risk of hospital re-encounters.

Our program had several key elements, including the use of full AUDITs for risk stratification and tailored intervention by social work-trained, bilingual SUNs. Patients with risky or hazardous use and lower AUDIT scores (< 12) respond well to brief interventions alone, which the SUNs provided per protocol.^49–51^ Due to constrained program resources, we deliberately prioritized more intensive support for patients likely to have AUD (AUDIT score ≥ 12), including consideration of MAUD to reduce harmful drinking.^52–54^ Among those with high AUDIT scores, over 75% had an alcohol-related admission. We leveraged hospitalization as a “reachable moment,” receiving 59.6% of screen-positive patients overall and achieving an almost 80% intervention engagement rate for patients we approached before discharge.^15,26^ This is comparable to or better than SBIRT uptake rates in prior studies.^55–58^

While studies have explored brief interventions and SBIRT/STIR for UAU in primary care and emergency settings,^17–23,49^ implementing these models in the hospital offers additional advantages, including easy incorporation due to intervention brevity, the flexibility to repeat sessions during hospitalization as needed, and the opportunity to reach underserved patients who may lack regular access to outpatient healthcare. Additionally, by incorporating structured linkage to outpatient care with a wide menu of recovery options, our highly trained social workers were able to deliver (at scale) many of the evidence-based features of addiction consult services. Addiction consult services have demonstrated increased enrollment and retention in outpatient treatment, decreases in short-term substance use, and reduced readmissions.^59–63^

We also achieved high rates of MAUD prescriptions of nearly 50%, far exceeding both national averages and prior single-center studies.^35–39,44,45^ Our project outcomes are more comparable to the performance of addiction consult services, for which recent retrospective studies have reported 30–40% MAUD initiation rates.^59,60^ Thus, even without dedicated addiction providers, implementation of this model has potential to improve clinical outcomes for patients with UAU.

### Limitations and Future Directions

As a pragmatic quality improvement initiative with constrained resources, our study has several limitations. Our screen-positive rate (6.0%) was lower than the estimated 15–20% hospital AUD prevalence reported in other studies.^9,13,64^ This may reflect implementation limitations of the electronic AUDIT-PC and may also reflect lower sensitivity during routine administration. Although self-administered AUDIT formats may reduce social desirability bias, we used oral administration to promote rapport with the SUN and to overcome possible literacy barriers.^65,66^

We were only able to reach around 30% of patients with likely AUD for phone follow-up assessments despite multiple attempts. Therefore, follow-up bias and reliance on self-reported adherence may overestimate intervention outcomes. We did attempt to confirm patients’ self-reports via chart review when able, though clinic notes were highly variable with whether and how MAUD adherence was documented. In the absence of a control group, improvements in AUDIT may also be overestimated due to regression to the mean and assessment effects.^65^ While the AUDIT was not originally designed to measure longitudinal change, it demonstrates adequate test–retest stability and is commonly used for this purpose.^48,66–68^ Differences in assessment time frames may limit direct comparability; however, the AUDIT’s focus on consequences of use, risk stratification, and reduced emphasis on precise consumption supports its utility for detecting meaningful changes in alcohol use behavior.^66–68^ We have not assessed follow-up beyond 3 months, so it is unclear whether these short-term improvements will be sustained. We were able to capture return visits within our multi-site hospital system, but did not have access to other hospital systems, so these rates may be undercounted. Due to limitations in follow-up data, we were unable to demonstrate whether MAUD initiation was associated with improved outcomes. A more complex analysis accounting for the presence and impact of other potential confounders, including co-morbid conditions, such as propensity scoring, or employing statistical methods to account for missing data, was beyond the scope of this pragmatic quality improvement intervention.

In the future, we have opportunities to add weekend staffing to increase patient reach. We plan to examine patient-defined and qualitative recovery goals to enrich our knowledge of recovery pathways, explore opportunities to increase medication adherence, observe long-term retention rates in treatment, tailor our intervention further to repeat clients, and obtain more robust follow-up metrics by utilizing community supports and personnel.

## CONCLUSIONS

In this quality improvement initiative, we implemented an inpatient screening and risk-stratified treatment protocol to identify and address UAU among hospitalized patients at a safety-net hospital, with high rates of MAUD initiation. Among patients reached for follow-up, we observed short-term changes in self-reported alcohol use and patterns of post-discharge care utilization.

## Supplementary Information

Below is the link to the electronic supplementary material.

ESM1 Appendix A: Description of Intervention Components and Implementation Details (DOCX 18.3 KB)

ESM2 Appendix B: Supplemental Data (DOCX 19.1 KB)

## Data Availability

The datasets generated during and/or analyzed during the current study are not publicly available due to confidentiality of individual patient data according to HIPAA laws and additional legal protections specific to patients with substance use disorders, but are available in a de-identified format from the corresponding author on reasonable request.
