# Supplementary material for: Implementation of a Hospital-Based Screening and Treatment Program for Unhealthy Alcohol Use
Source: J Gen Intern Med. 2026 May 14;41(14):4073–82. doi: 10.1007/s11606-026-10473-9 (PMC13728005; doi:10.1007/s11606-026-10473-9)
Supplement: Supplementary file 1 [file 11606_2026_10473_MOESM1_ESM.docx]

**Appendix A:** **Description of Intervention Components and Implementation Details**

***Substance Use Navigator (SUN) role and training***

SUNs with social work credentials were hired with program funding. SUNs were formally trained in an interactive workshop by a MINT-certified national expert in motivational interviewing. Patient engagement and successful delivery of the intervention was regularly interrogated and assessed at bi-monthly meetings with the MINT specialist and project team members.

The SUN’s received basic training and instruction on medications for alcohol use disorder (MAUD) during onboarding from two physicians with expertise in pharmacotherapy for AUD. Training consisted of a short didactic session on the indications and evidence basis behind MAUD, focusing on naltrexone. Handouts with clinical algorithms, shared decision-making aids, and pamphlets were provided. In addition, didactic presentations on AUD treatment were delivered to internal medicine trainees and faculty at the beginning of the project. Consultation via phone and secure text messaging with the project MDs was available to SUNs and these topics were discussed regularly in team meetings. While SUNs made recommendations, the decision to prescribe medications was made by the treating inpatient physician.

***Screening instruments and assessment***

Nurses completed the AUDIT-PC, a 5-question screening tool, in the electronic medical record (EMR) for every admitted patient. The AUDIT-PC is a shortened version of the 10-question Alcohol Use Disorders Identification Test (AUDIT), which is the most widely used and validated instrument to identify high-risk alcohol use.^48^

The electronic admission intake form – of which the AUDIT-PC is a component – is completed by nurses within 24 hours of a patient’s admission to the floor, but the electronic intake form is not completed in the emergency department where patients may have been held more than 24 hours due to unavailability of beds. In addition, some patients are initially unable to participate due to illness or encephalopathy.

For patients identified as intervention-appropriate, the SUN administered the full AUDIT to confirm and stratify the degree of unhealthy alcohol use (UAU). The AUDIT was administered verbally by the SUN – in person for the initial assessment and by phone for follow-up assessments.

***SUN identification and workflow***

On weekdays, SUNs reviewed a daily list of newly admitted patients that included admission diagnoses and completed AUDIT-PC scores. Patients were identified as intervention-appropriate based on positive AUDIT-PC scores (≥5) or alcohol-related admission diagnoses. Patients could also be referred to SUNs via direct consult.

After completing the intervention, the SUN documented the encounter in the EMR and recorded relevant data into a HIPAA-compliant Research Electronic Data Capture (REDCap) database. The SUN communicated with the patient’s outpatient care team via direct messaging in the EMR, when available, to provide handoff regarding the patient’s care plan.

***Brief intervention and referral approach***

For patients with AUDIT scores <12, who were considered at lower risk for moderate-to-severe AUD, the SUN completed a brief behavioral intervention (BI) consisting of motivational interviewing and counseling about alcohol use and placed a referral to primary care (SBIRT model). Given constrained resources and evidence supporting the effectiveness of BI in patients with lower-risk UAU, additional outreach and outcome measurement were not prioritized for this group.

The brief intervention was structured as a brief negotiated interview (BNI), an intervention designed to identify hazardous and harmful drinking, with the following steps:

1) ask permission to discuss alcohol use,

2) provide feedback on AUDIT score and guidelines on healthy drinking,

3) explore motivation to change alcohol use, and

4) discuss advice and next steps for addressing alcohol use.

For patients with AUDIT scores ≥12, who were considered at high risk for moderate-to-severe AUD, the SUN delivered a BI and primary care referral as above and additionally provided counseling regarding MAUD. If the patient expressed interest in medication and had no medical contraindications, the SUN recommended MAUD initiation to the inpatient care team prior to discharge (STIR model). The SUN also facilitated referrals as appropriate, including referral to the partner FQHC addiction clinic, rehabilitation facilities, Alcoholics Anonymous, peer recovery support, and sober living programs.

All patients were provided referral to appropriate follow-up care at discharge as able.

Workflow was flexible to accommodate the clinical demands of the SUN’s, but in general, new patients were seen and evaluated in the first part of the workday, and documentation with post-intervention assessments were completed subsequently. Our secure RedCap database maintained a real-time list of patients that were 4-6 weeks post-discharge and how many attempts had been made to contact them by phone. Patients were removed from this list once their outcomes assessment was complete, or once three attempts had been made to contact the patient with no response.

***Follow-up outreach and outcomes assessment***

At 4–6 weeks after discharge, the SUN attempted to contact patients by phone to complete an outcomes assessment. If the patient had consented to text messaging outreach, the SUN additionally attempted contact via text message. The SUN made three call attempts at three different times before marking the patient as “unable to contact.”

If reached, the SUN completed a follow-up AUDIT using a modified time frame of “time since discharge” and asked about outpatient appointment attendance, adherence to MAUD, interval emergency department visits or readmissions, and the need for additional resources or navigation.

***Chart review procedures***

For all patients with an AUDIT score ≥12, an inpatient chart review was completed to determine whether the patient had any emergency department encounters or hospital readmissions within 30 days of discharge from the index hospitalization, including encounters at other hospitals within the same health system.

For patients with AUDIT scores ≥12 who were referred to the partner FQHC clinic system, an outpatient chart review was attempted to determine attendance at follow-up appointments and medication adherence. If patients followed up at clinics outside the partner FQHC system, chart review visibility was limited.

***Statistical methods***

We analyzed the population of patients with positive AUDIT-PC screening and stratified patients by completion of the intervention, and further stratified patients who engaged by AUDIT score. Using odds ratios, we compared demographic characteristics between groups, including sex, age, race, housing status, and insurance status.

For patients who completed the intervention, we recorded relevant data about their hospital admission and intervention uptake, including full AUDIT score, medication eligibility and initiation, follow-up appointment date, and clinic information.

For patients with AUDIT ≥12 that were reached for follow-up assessment, we compared initial AUDIT scores to follow-up AUDIT scores and calculated the mean difference. A decrease was defined as at least a one-point decrease in score. We also determined medication adherence and appointment attendance. We used multivariate analysis to compare characteristics between patients who were and weren’t reached for follow up.

For patients with AUDIT ≥12, we conducted additional analyses for return encounters within 30 days. We compared demographics of those who did and did not have return encounters with odds ratios (adjusted and unadjusted), as well as the following characteristics: pre- and post- AUDIT scores, change in AUDIT score, medication initiation and adherence, and appointment attendance. We used logistic regression and simple t-test to compare between the groups. We also conducted multivariate analyses on predictors of return encounters.
