# Supplementary material for: Implementation of a Hospital-Based Screening and Treatment Program for Unhealthy Alcohol Use
Source: J Gen Intern Med. 2026 May 14;41(14):4073–82. doi: 10.1007/s11606-026-10473-9 (PMC13728005; doi:10.1007/s11606-026-10473-9)
Supplement: Supplementary file 2 [file 11606_2026_10473_MOESM2_ESM.docx]

**Appendix B: Supplemental Data**

**Appendix Table 1: Follow-up Appointment Referrals and Attendance**

|  | **Total** | **Attending follow-up** | **Did not attend follow-up** | **Unknown follow-up attendance** |
| --- | --- | --- | --- | --- |
| Total # of referrals made | 304 | 161 | 110 | 33 |
| CUC* | 215 (70.7%) | 126 | 77 | 12 |
| CareCo^†^ | 66 (21.7%) | 30 | 33 | 3 |
| Other | 23 (7.6%) | 5 | 0 | 18 |
| No referral made | 185 | 33 | 6 | 146 |
| Declined referral | 27 (14.6%) | 3 | 1 | 23 |
| Following with own PCP | 89 (48.1%) | 19 | 5 | 65 |
| Discharged to Skilled Nursing | 27 (14.6%) | 3 | 0 | 24 |
| Discharged to Rehabilitation | 32 (17.3%) | 8 | 0 | 24 |
| Other | 10 (5.4%) | 0 | 0 | 10 |

*CUC: CommUnity Care Clinic – FQHC

^†^CareCo: Care Connections Clinic – post-discharge clinic affiliated with CommUnity Care

**Appendix Table 2: Average reduction in AUDIT Score, by demographic characteristics**

|  | **Average AUDIT Score Change** |
| --- | --- |
| **Sex** | |
| Male | -14.2 |
| Female | -14.7 |
| **Age** | |
| < 50 years | -13.9 |
| ≥ 50 Years | -14.7 |
| **Self-identified Race** | |
| White or Caucasian | -15.6 |
| Hispanic or Latinx | -13.0 |
| Black or African American | -12.3 |
| Other or Unknown* | -14.9 |
| **Housing Status** | |
| Housing / Temporary Housing | -14.4 |
| No Housing | -12.8 |
| **Insurance Status** | |
| Commercial | -15.1 |
| Uninsured | -13.6 |
| MAP (Medical Access Program) | -14.1 |
| Medicaid | -12.0 |
| Medicare | -14.8 |
| Other or Unknown | -17.0 |
| **Admission Characteristics** | |
| Admitted due to alcohol use | -15.0 |
| Not admitted due to alcohol use | -11.9 |

*Other category includes the following: American-Indian, Asian, Native Hawaiian, and other.

**Appendix Table 3: Distribution of AUDIT score change in contacted patients**

| **AUDIT score change** | **# of patients** |
| --- | --- |
| Decrease of 20-30+ points | 38 (30.2%) |
| Decrease of 10-19 points | 53 (42.1%) |
| Decrease of 1-9 points | 19 (15.1%) |
| No change | 13 (10.3%) |
| Increase of 1-9 points | 2 (1.6%) |
| Increase of 10+ points | 1 (0.8%) |
| **Total** | **126** |
